# Persian version of frontal assessment battery: Correlations with formal measures of executive functioning and providing normative data for Persian population

**Published:** 2016-01-05

**Authors:** Sina Asaadi, Farzad Ashrafi, Mahmoud Omidbeigi, Zahra Nasiri, Hossein Pakdaman, Ali Amini-Harandi

**Affiliations:** 1 Functional Neurosurgery Research Center, Shahid Beheshti University of Medical Sciences, Tehran, Iran; 2 School of Medicine, Shahid Beheshti University of Medical Sciences, Tehran, Iran; 3 Department of Neurology, School of Medicine AND Loghman Hospital, Tehran University of Medical Sciences, Tehran, Iran

**Keywords:** Frontal Lobe, Executive Function, Outcome and Process Assessment, Mental Status, Persian, Iran

## Abstract

**Background:** Cognitive impairment in patients with Parkinson’s disease (PD) mainly involves executive function (EF). The frontal assessment battery (FAB) is an efficient tool for the assessment of EFs. The aims of this study were to determine the validity and reliability of the psychometric properties of the Persian version of FAB and assess its correlation with formal measures of EFs to provide normative data for the Persian version of FAB in patients with PD.

**Methods:** The study recruited 149 healthy participants and 49 patients with idiopathic PD. In PD patients, FAB results were compared to their performance on EF tests. Reliability analysis involved test-retest reliability and internal consistency, whereas validity analysis involved convergent validity approach. FAB scores compared in normal controls and in PD patients matched for age, education, and Mini-Mental State Examination (MMSE) score.

**Results:** In PD patients, FAB scores were significantly decreased compared to normal controls, and correlated with Stroop test and Wisconsin Card Sorting Test (WCST). In healthy subjects, FAB scores varied according to the age, education, and MMSE. In the FAB subtest analysis, the performances of PD patients were worse than the healthy participants on similarities, fluency tasks, and Luria’s motor series.

**Conclusion:** Persian version of FAB could be used as a reliable scale for the assessment of frontal lobe functions in Iranian patients with PD. Furthermore, normative data provided for the Persian version of this test improve the accuracy and confidence in the clinical application of the FAB.

## Introduction

Parkinson’s disease (PD) is a neurodegenerative disorder characterized by gradual impairment of affective, cognitive, and motor function.^1^ Although motor symptoms such as resting tremor, bradykinesia, rigidity, and postural instability are the hallmark of this disorder, cognitive, and psychiatric non-motor symptoms are equally disabling and directly impact the quality of in PD patients.^2^

Recent reports show that even after controlling for duration and severity of motor symptoms, cognitive abilities, such as executive and visuospatial functions, remain as the main problem in management of PD.^3^

Executive functions (EFs) consist of higher order processes including working memory, reasoning, task flexibility, and problem-solving as well as planning and execution.^4^^,^^5^ The identification of executive dysfunctions is useful for diagnosis and prognosis of brain diseases.^5^

Frontal assessment battery (FAB) was designed as a fast and efficient bedside battery to detect frontal lobe dysfunction in a variety of patients.^6^ FAB is divided into six subtests, each one assessing an “executive” function thought to be subserved by the frontal cortex.^4^

FAB has been largely used in several groups of patients such as Alzheimer’s disease,^7^ frontotemporal dementia,^8^ PD,^9^ Huntington’s disease,^10^ and other conditions.^11^

Oguro et al.^12^ have demonstrated the FAB sensitivity to differences in the executive dysfunction profiles of Alzheimer’s and vascular dementia patients (patients with vascular dementia had the worst performance).

Normative data have also been provided for healthy population samples. Studies showed that in healthy participates FAB were influenced by age and education (they were lower as age increased and education decreased).^13^^,^^14^

There is no information about the correlation of the FAB with formal measures of EF for screening executive dysfunctions in Iranian patients with idiopathic PD.

Aims of this study were to determine the validity and reliability of FAB in Iranian patients with PD and to establish normative data derived from a healthy sample of the Persian population.

## Materials and Methods

Among 76 patients diagnosed as idiopathic PD who were being followed up in the Shohada-e-Tajrish Hospital’s, Movement Disorders Clinic from 2012 to 2014, 49 patients (31 men and 18 women) were included in this study. PD diagnosis was made on the basis of the UK Brain Bank Criteria.^15^

The normative study involved 149 (86 men and 63 women) healthy subjects, who were among the caregivers of patients, and also among people who attended the hospital for a routine check-up.

All patients and controls were from various regions of Iran who were referred to our hospital. None of the participants had a current or past history of alcohol or drug abuse, current depression or psychiatric diseases, history of traumatic brain injury, neurological illness, or other reported conditions that could affect mental state as assessed by an individual clinical interview. 

Those who had < 5 years of education, those with Mini-Mental State Exam (MMSE)^16^ < 24, and other differential diagnoses of parkinsonism were excluded through neurologic examinations and radiologic evaluations.

Translation of FAB into Persian: FAB has been validated for PD,^5^^-^^17^ showing high correlation with classic frontal neuropsychological tests and significant differences between patients and controls.^18^^-^^20^ Functional brain imaging studies have shown significant correlation between FAB performance and perfusion in the medial and dorsolateral frontal cortex.^21^

FAB takes about 10 minutes to be administered and can be applied by any practitioner. It consists of six subtests: conceptualization (similarity), mental flexibility (fluency), motor programming (Luria motor series), sensitivity to interference (conflicting instructions), inhibitory control (Go-No-Go Task), and environmental autonomy (prehension behavior). Each subtest is scored between 0 and 3; a composite score ranging between 0 and 18 indicates whether or not executive dysfunction is present and, if yes, indicates the severity.

The FAB was adapted from the original English version into Persian.^5^ The battery was first translated independently by five persons with an advanced understanding of English. Different translations were combined by two independent experts, minor inconsistencies solved, and a preliminary version was produced. After that, the consensus version was then back-translated into English by another person fluent in the both languages and was then compared conceptually with the original text.

According to our pilot study, a linguistic adaptation was made in one of the subtests: the letter used in the original lexical fluency subtest, “S,” was replaced by “B,” which is as frequent in Farsi as “S” is in English. This is because in Persian language there are different words with “S” sound that might be confusing especially for people with lower levels of education. After reaching consistency for all verbal instructions and performing some pilot administrations, the final version of the Persian FAB was wrote.

Patients were tested in the “on” state when the medication minimizes or eliminates motor symptoms. After a brief clinical interview and collection of demographic features, unified PD Rating Scale,^22^ MMSE, Stroop test, Wisconsin Card Sorting Test (WCST),^23^ and the Persian version of FAB were applied to all patients.

Healthy participants were tested individually by a neurologist. As for PD patients demographic features, MMSE and the Persian version of FAB were administered. To determine the reliability of this study, the FAB results were compared with those of the Stroop test and WCST, which are sensitive to frontal lobe functions; and the frequently used the MMSE, which assesses the general cognitive functions. Internal consistency, inter-rater reliability (n = 28), and test-retest reliability (n = 29) were examined to test reliability of Persian version of FAB. Moreover, convergent validity was used in examining the validity of the Persian version of FAB. In the convergent validity approach, non-parametric correlations of simultaneously applied FAB and Stroop test, FAB and WCST and FAB and MMSE tests were calculated. It is expected that these comparisons would indicate a relationship because the Stroop test and WCST assess frontal lobe functions, as does the FAB.

This study was approved by the Shahid Beheshti University of Medical Sciences Ethics Committee, and patients included in this study gave their informed consent to participate.

The SPSS software (version 19, SPSS Inc., Chicago, IL, USA) was used for statistical analysis. Cronbach’s alpha was used to test the internal consistency, and the intraclass correlation coefficient was used for inter-judge reliability and test-retest reliability. In both tests, a score close to 1 indicates higher reliability. Pearson and Spearmen correlation tests were used for the correlation of FAB scores with age, education, and MMSE as appropriate. P < 0.050 were considered statistically significant.

## Results

The demographic features of patients with PD and healthy participants are shown in table 1. Internal consistencies of FAB scores in PD patients and in the control group are 0.68 and 0.53, respectively. High alpha values were obtained in both control groups and patients with PD. Statistically significant consistency [r = 0.89; 95% confidence interval (CI): 0.72-0.95] was observed in the test results conducted monthly. High intra-rater reliability rate was also found (r = 0.90). 

Concurrent validity was analyzed by calculating partial correlations between scores on the FAB, MMSE, WCST, and Stroop test (Table 2). Means and standard deviations (SD) of the total FAB scores stratified by age and education, for healthy participants are presented in table 3.

**Table 1:** Demographic features of Parkinson’s disease patients and healthy participants

**Variable**	**Groups**				**P**
	**PD (n = 49)**		**Healthy participants (n = 149)**		
	**Mean ± SD**	**Min-Max**	**Mean ± SD**	**Min-Max**	
Age	61.73 ± 9.13	39-80	59.32 ± 8.01	48-81	0.700
Education	9.65 ± 3.36	7-14	11.30 ± 2.23	7-14	0.510
Sex (Male/Female)	31/18		86/63		
FAB	12.96 ± 2.93	7-18	15.680 ± 1.701	13-18	< 0.001*
MMSE	28.02 ± 1.38	28-29	28.19 ± 1.47	25-30	< 0.001*
Disease duration	6.3 ± 3.1	1-15	0	0	-
UPDRS (I-III)	45.29 ± 18.62	10-96	0	0	-

FAB: Frontal assessment battery; MMSE: Mini-Mental State Examination; UPDRS: Unified Parkinson’s Disease Rating Scale; PD: Parkinson’s disease; SD: Standard deviation

* P < 0.050 significant

**Table 2:** Concurrent validity of frontal assessment battery, Stroop test, Wisconsin Card Sorting Test‎, and Mini-Mental State Examination

**Tests**	**Groups**	
	**PD patients (n = 49)**	**Healthy participants (n = 149)**
Stroop duration	-0.286*	-0.314*
Stroop error number	-0.384*	-0.280*
Stroop error correction	-0.405*	-0.385*
WCST number of categories	-0.373*	-0.271*
WCST perseverative errors	-0.408*	-0.324*
MMSE	-0.708*	-0.628*

WCST: Wisconsin Card Sorting Test; MMSE: Mini-Mental State Examination; PD: Parkinson’s disease

* Pearson correction coefficient (all comparisons are significant at level of P < 0.050)

**Table 3:** Mean total frontal assessment battery scores by age and education for the healthy sample

**Education (years) (mean ± SD)**	**Age (year)**			
	**30-49**	**50-69**	**70-89**	**Total**
1-4	14.1 ± 5.7	13.4 ± 3.4	12.3 ± 4.6	13.2 ± 1.4
5-8	14.8 ± 1.3	14.1 ± 2.4	13.2 ± 2.8	14.3 ± 2.1
9-12	16.1 ± 2.5	15.7 ± 2.1	14.9 ± 7.1	15.8 ± 6.4
> 12	16.7 ± 4.1	16.6 ± 6.7	15.8 ± 4.9	16.6 ± 3.4
Total	15.7 ± 6.2	14.6 ± 4.9	13.8 ± 4.7	15.0 ± 1.7

SD: Standard deviation

**Table 4:** Frequency distributions of the scores in the single subtests of the frontal assessment battery for the healthy participants (n = 149)

**Score**	**Subtests [n (%)]**					
	**Similarity**	**Fluency**	**Luria’s motor ** **series**	**Conflicting ** **instructions**	**Go-No-Go ** **task**	**Prehension behavior**
0	0 (0.0)	10 (6.7)	7 (4.6)	3 (2.0)	13 (8.7)	0 (0.0)
1	12 (8.0)	19 (12.8)	23 (15.4)	34 (22.8)	16 (10.7)	0 (0.0)
2	76 (51.0)	59 (39.6)	56 (37.5)	53 (35.5)	24 (16.1)	0 (0.0)
3	61 (40.9)	61 (40.9)	63 (42.3)	59 (39.5)	96 (64.4)	149 (100)

**Table 5:** Frequency distributions of the scores in the single subtests of the frontal assessment battery for Parkinson’s disease patients (n = 49)

**Score**	**Subtests [n (%)]**					
	**Similarity**	**Fluency**	**Luria’s motor ** **series**	**Conflicting ** **instructions**	**Go-No-Go ** **task**	**Prehension behavior**
0	7 (14.3)	9 (18.4)	1 (2.0)	4 (8.2)	2 (4.1)	0 (0.0)
1	10 (20.4)	19 (38.8)	2 (4.1)	6 (12.2)	7 (14.3)	0 (0.0)
2	26 (53.1)	16 (32.7)	9 (18.4)	18 (36.7)	19 (38.8)	0 (0.0)
3	6 (12.2)	5 (10.2)	37 (75.5)	21 (42.9)	21 (42.9)	149 (100)

A multiple regression analysis was performed to check the influence of demographic variables and MMSE score in normal participants. Total FAB scores were taken as the dependent variable and age, gender, education, and MMSE total score as independent variables. The resulting regression model excluded gender; age, education, and MMSE could explain 41.6% of the total variance of the FAB [R^2^ = 0.513; F_(4,143)_ = 28.42, P < 0.010]. There was a strong positive effect of education [coefficient = 0.489, t (143) = 4.85, P < 0.050], a negative effect of age [coefficient = −0.386, t (143) = −2.143, P < 0.010], and a positive effect of the MMSE score [coefficient = 0.708, t (143) = 2.43, P < 0.010]; thus, FAB results are lower in older and less educated subjects with lower MMSE scores.

Table 4 reports the frequency distribution of the scores in each FAB subtest. Three of them, i.e., similarities, fluency, and Luria motor series, were the most discriminative. By contrast, all subjects had the maximum possible on prehension behavior. The distribution of the subscores as well as of the total score is skewed toward higher values.

The performance of PD patients on the FAB subtests is shown in table 5. As for normal controls, a multiple regression analysis was calculated to check the influence of age, education, and MMSE on the total FAB scores. The regression model excluded gender, but it included age, education, and MMSE. It could explain 40.5% of the total variance [R^2^ = 0.375; F_(4,47)_ = 5.21, P < 0.002]. There was a negative effect of age [coefficient = −0.386, t(47) = −2.29, P < 0.030], a positive effect of the MMSE score [coefficient = 0.708, t(47) = 4.23, P < 0.040], and education [coefficient = 0.489, t(47) = 2.49, P < 0.030]. As with normal controls, mean FAB scores were negatively correlated with age and positively with education and MMSE.

Even after adjusting for age, education, and MMSE, which were entered as covariates in an ANCOVA, PD patients obtained lower total FAB scores than normal controls [PD patients = 12.8 vs. normal controls = 14.9; ANCOVA, F_(1,199)_ = 15.9, P < 0.010]. This result indicates the good discriminant validity of the FAB. 

Table 6 shows the mean values of FAB subtests in PD patients and healthy participants.

## Discussion

Impairments in EF and learning in PD patients are due to deficient dopaminergic input from the basal ganglia to the prefrontal cortex.^24^

Previous studies showed that there are independent parallel loop circuits between the basal ganglia, cerebral cortex, and thalamus.

**Table 6:** Mean values of frontal assessment battery subtests in Parkinson’s disease patients and healthy participants

**Subtests**	**Groups**		**P**
	**PD patients (n = 49)**	**Healthy participants (n = 149)**	
	**Mean ± SD**	**Mean ± SD**	
Similarity	1.63 ± 0.88	2.33 ± 0.62	< 0.050*
Fluency	1.35 ± 0.90	2.15 ± 0.88	< 0.050*
Luria’s motor series	2.67 ± 0.65	2.17 ± 0.65	< 0.050*
Conflicting instructions	2.14 ± 0.93	2.13 ± 0.83	0.920
Go-No-Go task	2.20 ± 0.84	2.47 ± 0.88	0.660
Prehension behavior	2.86 ± 0.50	2.94 ± 0.23	0.120

* P < 0.050 significant; SD: Standard deviation

This connection could affect by dopamine deficiency during PD.^25^

In the striatum, sensorimotor, cognitive, and limbic regions can be distinguished, based on their connections with the cerebral cortex.^26^^,^^27^ It has been suggested that dysfunction in the caudate nucleus connection with dorsolateral prefrontal cortex, and the lateral orbitofrontal cortex is contribute to the cognitive impairment in PD.^28^^,^^29^

EFs are among the most frequently described cognitive changes in patients with PD. These functions refer to principles of cognitive organization and mental processes involved in the changing situations of daily life.^30^^,^^31^

The FAB is a simple scale for the assessment of EFs, which has not yet been validated in Iran. This study examined the applicability of the FAB in Persian population with PD. FAB scores in PD patients were lower comparing to healthy participants. It indicates that this battery has good discriminant validity. Furthermore, significant correlations were obtained between results on the FAB and on the other measures of EFs, which indicate that the FAB has good concurrent validity. Good internal consistency, measured by Cronbach’s alpha in PD patients and healthy participants, was found as well.

This study found a positive correlation between MMSE and FAB scores in healthy individuals and in the patients with PD.

The study by Dubois et al.^6^ did not find high correlation between FAB and MMSE scores, but a positive correlation was reported by Kenangil et al.^32^ in patients with PD, by Kugo et al.^33^ in cases with dementia, by Tunçay et al.^34^ in patients with Alzheimer’s disease, Schizophrenia, and PD, by Castiglioni et al.^35^ in cases with Alzheimer’s disease and frontotemporal dementia, by Lima et al.^9^ in healthy individuals and cases with PD; and by Beato et al.^36^ in healthy individuals. This could be explained by the weak distinctiveness and discriminant validity of FAB as well as the assessment of some frontal functions through MMSE. However, the relationship of the cognitive functions of FAB with general measurements and further data, such as sub-group analysis of MMSE, are required to clarify the relationship between FAB and MMSE.

In some previous studies age and educational level did not have any effect on the FAB scores.^5^^-^^33^ However in others and in the present study, FAB scores in healthy participants and participants with PD were found to be positively correlated with education and negatively correlated with age.^9^^-^^38^ In our study, FAB score in healthy participants and PD patients were found positively correlated with education level and negatively correlated with age. These findings support those of previous studies and emphasize the importance of these two factors in neurocognitive assessment.

For example, in the study by Kugo et al.,^33^ they found the higher FAB scores in comparison with the control group of Mok et al.’s study.^37^ One reason of this result may be the relatively low educational level and advanced age of the Mok et al. group.^37^

Another probable reason of differing results may be linguistic and cultural differences. For example, FAB scores that reported in the control cases of Kenangil et al.^32^ and Lima et al.^9^ were lower than FAB score in the control group of original study that was conducted in French.^5^

It was also observed that the FAB subtests are not equally discriminative, the finer ones being similarities, fluency, and Luria’s motor series (Table 6). Similarities and fluency were also found to be the most discriminative by Appollonio et al.^13^ The least discriminative was prehension behavior, a subtest that aims to evaluate environmental autonomy. This subtest has rarely elicited a score lower than 3 in healthy participants, and in clinical groups such as Alzheimer’s disease.^12^^-^^14^

Another goal of this study was to establish the concurrent validity of Persian version of FAB for use with Iranian PD patients. FAB scores were significantly correlated with a number of categories and perseverative errors in the WCST, Stroop error correction, and Stroop error number. These correlations strongly indicate that the Persian version of FAB does measure EF, and it has good concurrent validity.

## Conclusion

FAB scores of the PD patients were lower comparing to the healthy population. This study concludes that the Persian version of FAB could be used as a reliable scale for the assessment of frontal lobe functions, giving helpful information for the diagnosis of this disease and for evaluation of cognitive decline in Iranian patients with PD. Furthermore, we provide a normative data for Iranian healthy populations that improve accuracy and confidence in the clinical use of the FAB.

## Notes:

**How to cite this article:** Asaadi S, Ashrafi F, Omidbeigi M, Nasiri Z, Pakdaman H, Amini-Harandi A. Persian version of frontal assessment battery: Correlations with formal measures of executive functioning and providing normative data for Persian population. Iran J Neurol 2016; 15(1): 16-22.
